# Local amplification of glucocorticoids in the aging brain and impaired spatial memory

**DOI:** 10.3389/fnagi.2012.00024

**Published:** 2012-08-29

**Authors:** Joyce L. W. Yau, Jonathan R. Seckl

**Affiliations:** Centre for Cognitive Ageing and Cognitive Epidemiology and Endocrinology Unit, Centre for Cardiovascular Science, The Queen's Medical Research Institute, University of EdinburghEdinburgh, UK

**Keywords:** corticosterone, cortisol, watermaze, hippocampus, 11β-HSD1, neurosteroids, CYP7B1

## Abstract

The hippocampus is a prime target for glucocorticoids (GCs) and a brain structure particularly vulnerable to aging. Prolonged exposure to excess GCs compromises hippocampal electrophysiology, structure, and function. Blood GC levels tend to increase with aging and correlate with impaired spatial memory in aging rodents and humans. The magnitude of GC action within tissues depends not only on levels of steroid hormone that enter the cells from the periphery and the density of intracellular receptors but also on the local metabolism of GCs by 11β-hydroxysteroid dehydrogenases (11β-HSD). The predominant isozyme in the adult brain, 11β-HSD1, locally regenerates active GCs from inert 11-keto forms thus amplifying GC levels within specific target cells including in the hippocampus and cortex. Aging associates with elevated hippocampal and neocortical 11β-HSD1 and impaired spatial learning while deficiency of 11β-HSD1 in knockout (KO) mice prevents the emergence of cognitive decline with age. Furthermore, short-term pharmacological inhibition of 11β-HSD1 in already aged mice reverses spatial memory impairments. Here, we review research findings that support a key role for GCs with special emphasis on their intracellular regulation by 11β-HSD1 in the emergence of spatial memory deficits with aging, and discuss the use of 11β-HSD1 inhibitors as a promising novel treatment in ameliorating/improving age-related memory impairments.

## Introduction

Cognitive decline is a key feature of aging but significant impairments of learning and memory are not inevitable or strictly linked to chronological age. Marked inter-individual variability exists, ranging from almost no decline through mild impairments to frank dementia. This phenomenon has been described in several species including rodents and humans but the mechanisms underlying the individual differences remain poorly understood. One important mechanistic hypothesis is that variations in hypothalamic-pituitary-adrenal (HPA) activity and consequent exposure to glucocorticoids (GCs; cortisol in humans, corticosterone in rodents) during life may contribute to the inter-individual differences in cognitive decline in animals and humans.

The adrenal cortex synthesizes GCs and these steroid hormones are released directly into the peripheral circulation following stimulation of the HPA axis in response to external (stress) and internal (circadian) cues. Collectively, GCs released in coordination with the rapidly acting sympathetic-adrenomedullary system, help an organism respond to “stressors” or threats to homeostasis by mobilizing energy stores, suppressing nonessential physiological processes (e.g., reproduction, digestion) and initiating behavioral responses. Circulating GC levels are normally tightly regulated by negative feedback inhibition upon the HPA axis where GCs act back on the hypothalamus and pituitary glucocorticoid receptors (GRs) (to suppress CRH and ACTH production) to terminate its own release. GC feedback also occurs in higher centers such as the hippocampus and cingulate cortex.

GCs readily enter the brain, a major target for GC action (McEwen et al., 1968). Here GCs bind to classical nuclear (hormone) receptors to regulate the transcription of specific genes, either by direct binding of receptor homodimers to DNA (Datson et al., 2001) or via protein-protein interactions with other transcription factors such as fos and Jun-1 (Heck et al., 1994; Hayashi et al., 2004). Through activation of their intracellular receptors, GCs affect a wide range of processes including altering neurotransmission, electrophysiological activity, cellular metabolism, and structure, as well as neuronal division, maturation, and death.

### Corticosteroid receptors

**Figure 1:**
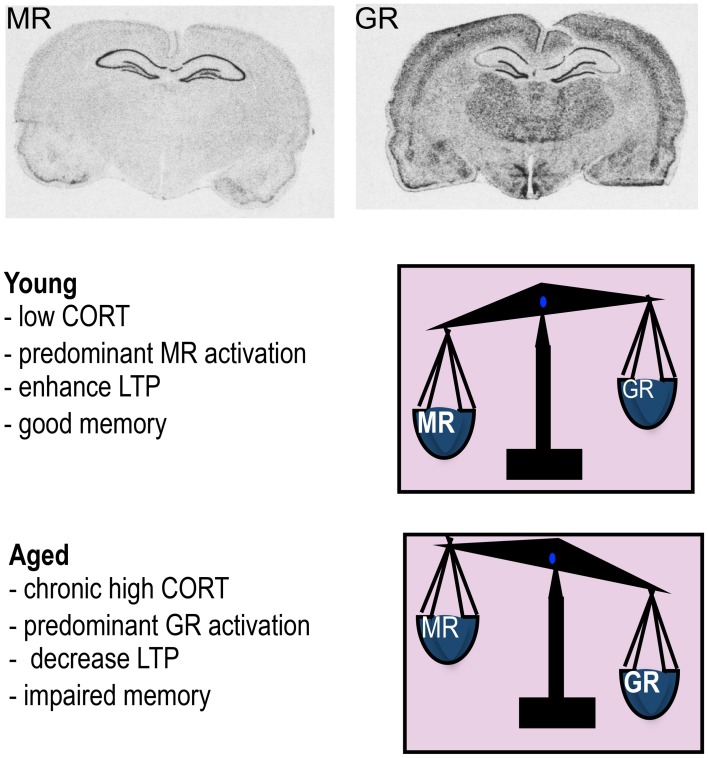
**Mineralocorticoid receptor (MR) and glucocorticoid receptor (GR) mRNA expression in rat brain.** The high affinity MR is predominantly occupied under low corticosterone (CORT) levels typically observed in young animals under basal conditions and this leads to enhanced LTP and good memory. When CORT levels rises such as in aging, MR is fully occupied and the excess GCs now occupy more GR so the balance of receptors activated is now predominantly GR which decreases LTP and impairs memory.

Two types of corticosteroid receptors exist, the type I high affinity mineralocorticoid receptors (MR) and type II lower affinity GRs (Reul and de Kloet, 1985). Although sharing almost identical DNA-binding domains, MR and GR can exert distinct cellular functions as the genes they bind to show little overlap (Datson et al., 2001). GRs are widely distributed throughout the brain in most neurons and glia. GRs have a lower affinity for physiological GCs and only become substantially activated as hormone levels rise following stress. MR is expressed in neurons only and has a more restricted distribution with high expression confined particularly to the hippocampus (Figure 1), and septum. MRs having a 10-fold higher affinity for physiological corticosteroids (GCs and the mineralocorticoid aldosterone) are extensively occupied under basal conditions when hormone levels are low (Reul and de Kloet, 1985; McEwen et al., 1986).

**Figure 2:**
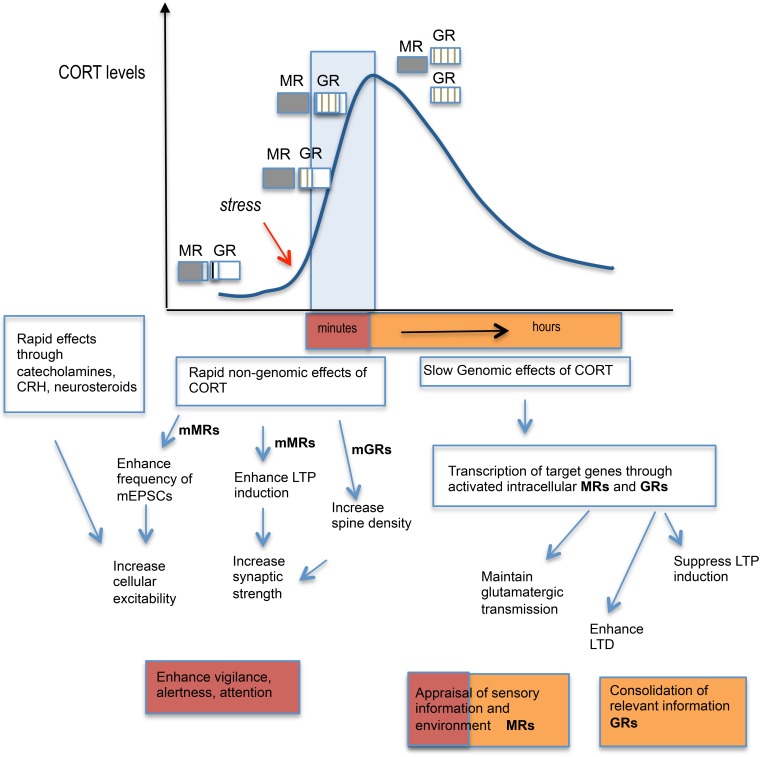
**Schematic diagram showing stress hormone effects: from cellular responses to behavior.** Activation of the HPA axis by stress leads to a rapid rise in circulating corticosterone (CORT) levels which starts to fully occupy MR and increasingly occupy GRs. The varying level of occupancy of MRs and GRs are represented by extent of shading or lines within the boxes. The CORT levels normally return to baseline levels by 2 h through negative feedback regulation upon the HPA axis. The initial rapid effects of CORT via activation of membrane-located MRs enhance LTP induction and enhance miniature excitatory postsynaptic currents in the hippocampus while membrane-located GRs appear to increase neuronal spine density. Together with other stress hormones released such as the catecholamines (noradrenaline and adrenaline) and corticotropin-releasing hormone (CRH), which also strengthen synaptic transmission in the hippocampus, these rapid events contribute to enhanced vigilance, alertness, and attention. The slower genomic effects of CORT through activation of intracellular MRs and GRs and the transcription of target genes, leads to the maintenance of glutamatergic transmission via MRs and suppression of LTP induction but enhanced LTD via GRs in the hippocampus. MRs and GRs contributes to different aspects of cognitive processing with MR activation responsible for the appraisal of sensory information and environmental cues, while GRs are important for the consolidation of events/information in memory.

In addition to the delayed genomic effects via intracellular MR and GR, it has become evident from recent work that GCs also affect brain function through rapid non-genomic membrane-associated mechanisms (Groeneweg et al., 2011) (Figure 2). The latter mode of action explains the rapid (minutes) effects of GCs on the excitability and activation of neurons in several brain regions (e.g., hypothalamus, hippocampus, amygdala, and prefrontal cortex) and provides a physiological basis for rapid effects on behavior (de Kloet et al., 2008). A membrane-localized form of MR appears to mediate the rapid GC signaling in the hippocampus (Karst et al., 2005). However, not all rapid GC effects occur via MRs with some [e.g., increase in spine density of hippocampal neurons (Komatsuzaki et al., 2005)] depending on membrane-located GRs rather than MRs and others [e.g., long-term potentiation (LTP) induction (Wiegert et al., 2006), NMDA-dependent neurotoxicity (Xiao et al., 2010)] occurring independent of MR or GR but mediated possibly through as yet unidentified membrane-localized receptors.

### Blood glucocorticoid levels

Short term increases in GC levels are normally adaptive and beneficial but prolonged exposure to elevated GC levels such as during chronic stress or as a consequence of failure or impairment of negative feedback control of GC secretion (e.g., Cushing's syndrome, major depression) will lead to excessive GC responses and to pathology in the periphery (e.g., diabetes, hypertension, osteoporosis, central obesity) and CNS (e.g., depression, impaired learning, and memory). Cushing's syndrome patients with hyper-secretion of cortisol show reduced hippocampal volume and impaired performance on hippocampal learning tasks (Starkman et al., 1992). Notably, successful treatment to correct the excessive secretion of GCs reverses the pathology including hippocampal structural recovery and the restoration, to some extent, of mood, learning, and memory (Starkman et al., 1999). Conversely, severely reduced GC levels, as in Addison's disease, also result in pathology and cognitive impairments (Tytherleigh et al., 2004) and are treated with lifelong corticosteroid replacement. Thus both too little and too much GCs can have detrimental effects on memory, emphasizing the crucial need to maintain optimal levels of GCs for health and survival.

## Glucocorticoids and hippocampus-dependent memory

GCs via binding to abundant MRs and GRs in the hippocampus control the excitability of neuronal networks that underlie learning and memory processes. While not the focus of this review, it is worth noting that other brain areas rich in GRs such as the basolateral amygdala (BLA) and prefrontal cortex, which controls emotional and working memory respectively, interacts with the hippocampus to modulate cognitive function (Roozendaal and McGaugh, 1997, 2011). An efficient interplay between activation of MR and GR appears essential for maximal learning. The two receptors have different roles in learning as evident from rodent studies, especially in the watermaze using antagonists of MR and GR at the various phases of spatial learning (Oitzl and de Kloet, 1992); MRs have a major role in behavioral reactivity toward stimuli while GRs are involved in consolidating learned information. Furthermore, the ratio of occupation and presumably activation of MR/GR appears to determine whether GCs improve or impair memory (de Kloet et al., 1999). Thus, optimal enhanced memory occurs when GC levels are mildly elevated such that most MRs and only some GRs are activated (i.e., increased MR/GR ratio) but impaired memory results when circulating GC levels are greatly increased such as found in some aging individuals (i.e., low MR/GR ratio) (Kim and Diamond, 2002; Tytherleigh et al., 2004; Herbert et al., 2006) (Figure 1). Indeed, circulating basal (nadir) GC levels do not always increase as a function of chronological age with only a proportion of individuals showing increasingly high GC levels with advancing age while some show levels within the young normal range (Lupien et al., 1994).

### MR and GR activation

Whether the consequence of the receptor activation is positive or negative for memory depend largely on the context of the situation/event to be remembered, the timing and the magnitude of the increased GC levels (de Kloet et al., 1999; Joels et al., 2006). Acute stress or increased GC levels occurring around the time of learning and within the context of the event to be remembered enhances memory consolidation. In contrast, they impair memory if occurring either before or a considerable time after the learning task (Quervain et al., 1998; Cazakoff et al., 2010). Evidence, mainly from animal studies, suggests that GCs preferentially enhances memory consolidation of emotionally arousing experiences (Roozendaal et al., 2006). Animal learning tasks, including the Morris watermaze and radial arm maze for spatial memory training, are designed to be generally affectively arousing because they require motivation to elicit changes in behavior. Even learning tasks that include no rewarding or aversive stimulation, such as the object recognition task, induces modest novelty-induced stress or arousal during training (Okuda et al., 2004). GCs released during learning appear essential for establishing enduring memories (De Kloet et al., 1998). Thus, small increases in GCs enhance hippocampus-mediated learning and memory while larger, prolonged elevations impair memory (Lupien and McEwen, 1997; Kim and Diamond, 2002). This follows the inverted U-shaped dose-response relationship between GC levels and effects on hippocampal LTP, an electrophysiological phenomenon associated with synaptic strengthening which is one of the major cellular mechanisms underlying learning and memory. Low moderate GC levels occupy predominantly high affinity MR which increases LTP and memory, while high GC levels occupy the lower affinity GR (in addition to MR) and impair LTP and memory (Pavlides et al., 1995, 1996; Kim and Diamond, 2002; Kim et al., 2007). One recent study supports the inverted-U-shaped relationship between intrinsic stress intensity (i.e., increased endogenous GCs induced by factors associated with the learning task, in this case water temperature) and spatial memory in the radial arm watermaze. Thus, rats trained at 19°C made fewer errors than rats trained at either more (16°C) or less (25°C) stressful conditions (Salehi et al., 2010).

### The aging hippocampus

The hippocampus not only plays a central role in the processing of spatial and contextual information (Morris et al., 1982; Moser et al., 1995) but also exerts an inhibitory influence over HPA function (Jacobson and Sapolsky, 1991). With its high density of MRs and GRs, the hippocampus is also particularly sensitive to the deleterious actions of chronic GC excess, potentiating neurotoxicity, dendritic atrophy, and perhaps even neuronal loss (Sapolsky, 1987). The idea that excess GCs could promote aging of the hippocampus was first established over 30 years ago following a study that showed a positive correlation between hippocampal aging (astrocyte reactivity as a marker of neuronal damage) and plasma levels of corticosterone in aging rats (Landfield et al., 1978). While only a few studies have shown high GC levels or stress actually cause hippocampal neuron loss (Uno et al., 1989; Sousa et al., 1998), much evidence support chronic stress (or high GCs) causing hippocampal atrophy (Watanabe et al., 1992; Magarinos and McEwen, 1995).

In humans, including those with Cushing's disease, Alzheimer's disease, depression, and normal aging, higher cortisol levels have been associated with poorer memory and hippocampal shrinkage/neuronal loss (De Leon et al., 1988; Wolkowitz et al., 1990; Newcomer et al., 1994; Mitchell and Dening, 1996; Lupien et al., 1998; Karlamangla et al., 2005; MacLullich et al., 2005). Moreover, increased HPA activity, as a consequence of impaired HPA axis negative feedback control, has been hypothesized to contribute to the decline in cognitive function, including deficits on spatial tasks with aging (Ohta, 1981; Lupien et al., 1998; McEwen et al., 1999). In support, the extent of age-related cognitive impairments in rodents and humans correlates with increased HPA activity (Issa et al., 1990; Meaney et al., 1995; Yau et al., 1995; Lupien et al., 1996, 1998). Aging rodents also show difficulty with hippocampus-dependent tasks that require a spatial mapping strategy; acquisition deficits have been reported in spatial information processing tests including Barnes hole-board task, radial arm mazes, and Morris watermaze (Ingram et al., 1981; Barnes and McNaughton, 1985; Gage et al., 1989; Issa et al., 1990; Gallagher et al., 1993; Yau et al., 1994, 1995).

### Glucocorticoids and other brain sites

Although GCs act on the hippocampus to modulate the formation of new memories, it also acts at other sites, notably the amygdala and prefrontal cortex, which functionally interact. Thus, GC effects on hippocampal LTP and memory can be blocked by lesions to the BLA (Roozendaal and McGaugh, 1997; Kim et al., 2001) and much evidence supports a role of the BLA via emotional arousal-induced noradrenergic activation and GR actions in the modulation of memory consolidation and working memory (Roozendaal and McGaugh, 2011). Given that amygdala function is affected by aging (Iidaka et al., 2002; Charles et al., 2003), it might be expected that fear conditioning would also be disrupted with aging and hence influence hippocampal memory deficits. However, age-related impairments in spatial memory appear not to be influenced by emotional and contextual memories, which tend to be preserved with aging (Comblain et al., 2004; Gould and Feiro, 2005; May et al., 2005; Bergado et al., 2011; Broster et al., 2012).

## Glucocorticoids and inter-individual differences in spatial memory

### Aged rats

**Figure 3:**
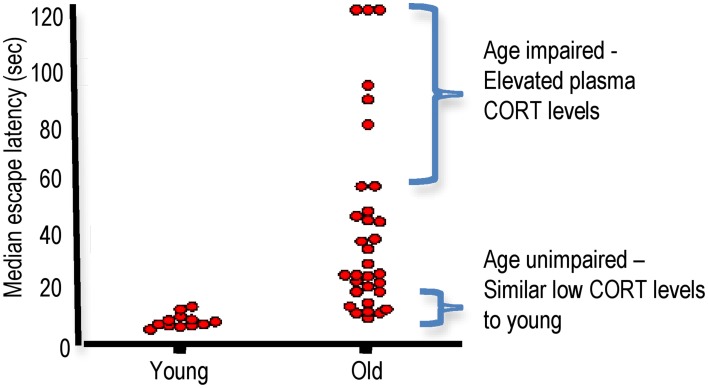
**Aged Lister hooded rats show inter-individual differences in spatial learning in the watermaze.** 24 months old male Lister hooded rats show impaired spatial learning and memory in the watermaze as a group compared to 6 months old controls. However, individual performances taking the median of the escape latencies (4 trials/day) for the last 2 days of training shows a large spread of learning abilities for aged rats. Plasma corticosterone (CORT) levels in the aged rats correlated negatively with spatial memory with impaired aged rats showing increased CORT while unimpaired aged rats had lower CORT similar to young controls (Based on Yau et al., 1995, with permission from Elsevier).

As a group, aged rats show impaired spatial learning. However, there are substantial inter-individual differences in performance (Yau et al., 1995). This increased variation in aged rats allows subdivision into categories (cognitively-unimpaired, cognitively-impaired) in the watermaze according to their latency to find the submerged platform on the last days of training relative to the mean latency of young controls (Issa et al., 1990; Tombaugh et al., 2002) or to a learning index score computed from probe trials (during retraction of the platform to the bottom of the pool during 30 s of 90 s trials) (Gallagher et al., 1993; Wilson et al., 2004; Robitsek et al., 2008). Aged rats have also been subdivided into impaired and unimpaired categories according to their spatial memory performance in a Y-maze two-trial spatial recognition task where the mean value of the percentage of visits in the novel arm of impaired rats were not different from chance values (Vallee et al., 1997). We typically find approximately ~20–25% of aged Lister hooded rat cohorts differ significantly (>2.5 SD) from young controls in the watermaze (escape latency and probe times) and another ~20–25% not significantly different (<0.5 SD) from young controls (Figure 3). It is the cognitively impaired groups that selectively show elevated plasma corticosterone levels (Issa et al., 1990; Yau et al., 1995), and reduced hippocampal corticosteroid receptor density (Issa et al., 1990; Yau et al., 1995) compared to both young and similarly aged cognitively unimpaired rats (Issa et al., 1990). While aged rats with watermaze performances not significantly different from young rats have been sub-classed as “cognitively unimpaired,” it is important to note that they are not cognitively the same as young rats. Indeed, we found that aged Lister hooded rats, categorized as unimpaired by their performance in the conventional spatial memory watermaze task, were impaired in a more cognitively demanding delayed matching-to-place paradigm spatial memory watermaze task (Steele and Morris, 1999) when compared to young controls (Yau and Seckl, unpublished).

### Aged mice

Aged mice also show impaired spatial memory in the watermaze (Verbitsky et al., 2004; Pawlowski et al., 2009) and increased plasma CORT levels (Yau et al., 2001, 2007; Holmes et al., 2010) such that higher CORT levels correlates with impaired learning in the watermaze (Yau et al., 2001). However, in contrast to aged rats, subdividing aged mice cohorts into cognitively aged-impaired and aged-unimpaired groups is less clear-cut with large variations in performance found even in young controls (Pawlowski et al., 2009). Mice in general take longer to train in the watermaze than rats and show less consistent performance with some aged mice tending to float rather than swim. The Y-maze spatial recognition task, in contrast to the watermaze, is an ethologically relevant test based on the rodent's innate curiosity to explore novel areas and presents no negative or positive reinforcers and little stress for the rodents. When spatial memory was tested in the Y-maze using a 2 h inter-trial interval, young C57BL/6J mice can still remember the novel arm (exploring it more than the other two arms) while aged mice were overall impaired as a group (not distinguishing the novel arm between the three arms). As in the watermaze, inter-individual differences in Y-maze spatial memory performances exist in aged mice, but with a smaller percentage of aged mice “unimpaired” (<20%) while the majority were “impaired” (Yau and Seckl, unpublished).

## Glucocorticoids and maintainence of spatial memory with aging

In rats, manipulations which keep GC levels low throughout life, such as postnatal handling during the first two weeks of life (which permanently increases hippocampal expression of GR, thus improving HPA axis negative feedback to reduce circulating GC levels), denser maternal care (licking and arched back nursing of her offspring), or adrenalectomy at middle age with low dose corticosterone replacement, prevent later hippocampal morphological changes and spatial memory deficits with aging (Landfield et al., 1981; Meaney et al., 1988). Although such manipulations are probably not clinically utilizable, they suggest that pharmacological treatments to increase GR density in the adult hippocampus may reduce GC levels long-term and ameliorate or prevent the emergence of spatial memory impairments with aging. One potent long-term regulator of MR and GR in the hippocampus is serotonin; this neurotransmitter directly increases GR in primary neuronal cultures and *in vivo* following postnatal handling of rat pups (Mitchell et al., 1990; Meaney et al., 1994; Lai et al., 2003) while lesions of the serotonergic pathway reduces hippocampal MR and GR (Yau et al., 1997). Antidepressants, which amongst other effects, increases serotonin levels, increase hippocampal GR density, improve HPA feedback regulation and thus reduce GC levels in adult rats and mice (Reul et al., 1993; Montkowski et al., 1995; Barden, 1996). Chronic (2 months) treatment of aged Lister hooded rats with amitriptyline, however, did not prevent spatial memory impairments but treatment of young (8 months) animals improved spatial memory, reduced plasma corticosterone levels, and increased hippocampal MR mRNA expression (Yau et al., 1995). Since hippocampal MR enhances LTP (Pavlides et al., 1994) and has a positive influence on memory while central MR blockade impairs spatial memory in adult rats (Yau et al., 1999), the antidepressant induced increase in hippocampal MR may, in part, underlie the better spatial memory in the young rats. Aged rats may lack the plasticity for antidepressants to be effective at enhancing memory later in life. In support, earlier treatment with antidepressants from middle age (for 6 months) improved HPA negative feedback (Rowe et al., 1997) and reduced the emergence of spatial memory impairments in a cohort of aged rats (Yau et al., 2002).

## Tissue selective regulation of glucocorticoid exposure

While many studies have measured blood GC levels and correlated this to GC actions within tissues of interest, the principal determinant of GC action is the level of hormone inside the cell. The magnitude of intracellular GC action has long been thought to be determined by the concentration of active hormone in the circulation [modulated by hormone binding to plasma proteins, mainly corticosteroid binding globulin (CBG)] and the density of intracellular receptors in target tissues. However, during the past two decades, enzymic pre-receptor metabolism of GCs by 11β-hydroxysteroid dehydrogenases (11β-HSDs) has emerged as a key mechanism for tissue specific control of active GC levels (Seckl, 1997). 11β-HSDs are microsomal (endoplasmic reticulum) enzymes which catalyse the interconversion of active GCs (corticosterone in rodents, cortisol in humans) and inert 11-keto forms [11-dehydrocorticosterone (11-DHC), cortisone]. They thus, potently regulate steroid access to receptors within specific tissues (Seckl, 1997).

### 11β-hydroxysteroid dehydrogenase type 1

**Figure 4:**
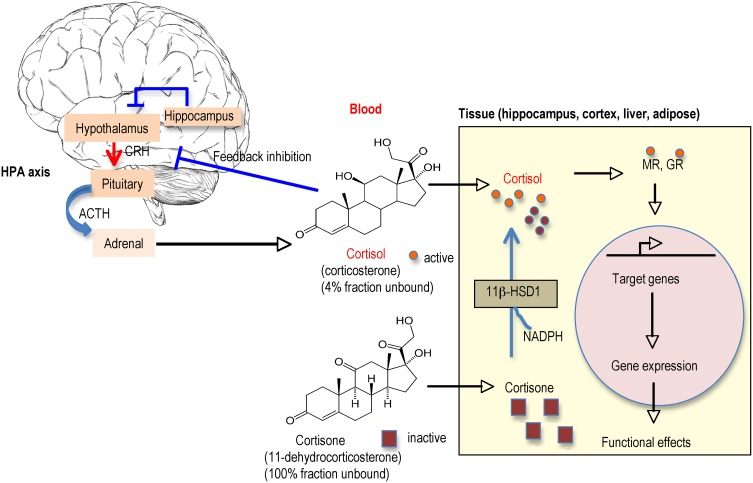
**Regulation of glucocorticoid (GC) levels in blood and tissues.** Circulating GC levels are tightly regulated by negative feedback inhibition at the hypothalamic-pituitary-adrenal (HPA) axis. Active GCs (cortisol in humans, corticosterone in rodents) circulate mostly bound to CBG (corticosteroid binding globulin) such that only ~4% are free to enter tissues. The inactive GCs (cortisone, 11-dehydrocorticosterone) the products of 11β-HSD2 metabolism mainly in kidney, circulates unbound to plasma proteins so all are available to enter tissues. Once inside the cell, there is another level of control by the presence of 11β-HSD1 (found in specific cells in the adult brain such as in hippocampus and cortex and other tissues such as liver and adipose). 11β-HSD1 regenerates active GCs from their inactive forms thereby effectively amplifying intracellular GC levels before they bind to MR and/or GR (depending on brain region). The activated receptors then translocate into the nucleus to activate the transcription of target genes.

11β-HSD1 is the predominant isoform in the adult rodent and human brain, where it is widely distributed with particularly high expression in the hippocampus, cerebellum, and cortex in both neurons and glia cells (Moisan et al., 1990; Sandeep et al., 2004). 11β-HSD2 whilst highly expressed in the fetal CNS until mid-gestation, in the adult brain is restricted to the nucleus of the solitary tract (NTS) in mice and this plus a few other scattered brain stem and diencephalic nuclei in rats. 11β-HSD2 acts as a dehydrogenase to inactivate GCs before they can bind to receptors. It is best noted for its role to exclude GCs from otherwise non-selective MRs in the distal nephron, thus allowing aldosterone selectivity. 11β-HSD1 in contrast functions as a 11β-reductase (regenerating active GCs from inert 11-DHC) in intact cells, thus locally “amplifying” GC levels within brain cells as well as in liver, adipose tissue, immune system cells etc (Figure 4). This direction of action, far from protecting neurons against GC excess, would be anticipated to increase local intraneuronal GC levels, potentiating their effects including toxicity. Consistent with this hypothesis, *in vitro* otherwise inert 11-DHC potentiates kainate neurotoxicity in hippocampal cells in culture, as a consequence of its conversion to active corticosterone by 11β-HSD1 expressed in the neurons, an effect lost in the presence of an 11β-HSD inhibitor (Rajan et al., 1996). The importance of such regeneration of GCs within cells *in vivo* was shown in mice homozygous for targeted disruption of the 11β-HSD1 gene (Kotelevtsev et al., 1997). 11β-HSD1 appears to be the only 11β-reductase, at least in mice, since knockout (KO) animals cannot convert 11-DHC to active corticosterone and showed evidence of reduced tissue GC actions (e.g., resist hyperglycaemia induced by obesity or stress) (Kotelevtsev et al., 1997). So are there brain effects of 11β-HSD1 deficiency?

### 11β-HSD1 and HPA axis activity

Expression of 11β-HSD1 in brain sites (prefrontal cortex, hippocampus, hypothalamus) and pituitary that underpin negative feedback actions of GCs suggests that this enzyme may influence HPA axis activity. In order to maintain tissue GC levels in feedback sites that normally express 11β-HSD1, higher levels of plasma GCs would be predicted to result as a consequence of loss of local production of active GCs at these sites. Indeed mice lacking 11β-HSD1 on the 129/MF1 strain showed evidence of reduced HPA axis feedback sensitivity (elevated nadir levels of plasma corticosterone, enlarged adrenal glands, and exaggerated GC response to an acute stressor) (Harris et al., 2001). However, the effects of 11ß-HSD1 deficiency on HPA axis activity were lost when the mice were bred onto another genetic background strain. Thus, 11β-HSD1 KO mice congenic on a C57BL/6J background show normal nadir plasma corticosterone levels and efficient negative feedback regulation thought to be due to compensatory increased GR expression in the hippocampus and paraventricular nucleus of the hypothalamus (Carter et al., 2009). Activation of the HPA axis is therefore not an inevitable consequence of 11β-HSD1 deficiency or inhibition. The genetic background appears crucial in governing the HPA axis response to 11β-HSD1 deficiency or inhibition.

## Implications of 11β-HSD1 function in the aging brain

If 11β-HSD1 locally regenerates active GCs thus amplifying GC action within specific cells *in vivo*, this direction of enzyme activity would be anticipated to impair spatial memory in the aging brain when there is the additional contribution of increased “free” unbound GCs from the periphery in individuals with elevated blood GC levels. Indeed, a lack of the enzyme in the aged brain appears beneficial to memory processes as shown in 11β-HSD1 KO mice (Yau et al., 2001). Thus, aged 11β-HSD1 KO mice congenic on the 129 strain, despite modestly elevated plasma corticosterone levels throughout life, show ameliorated GC-related learning impairments in the watermaze (Yau et al., 2001). *This is the opposite of what would be anticipated if only circulating corticosterone levels were taken into consideration*. Hippocampal tissue corticosterone levels measured *ex-vivo* were significantly lower in aged 11β-HSD1 KO mice than aged controls (Yau et al., 2001, 2011), supporting the important role the enzyme plays in determining the levels of GCs within cells and thus their action.

### Aged 11β-HSD1 knockout mice

Different genetic background mouse strains can exhibit marked differences in learning and memory (Owen et al., 1997). The original 11β-HSD1 KO mice generated on the 129 Ola background learnt a cued version of a watermaze task (Yau et al., 2001), but had difficulty learning the classical task. Subsequently, aged 11β-HSD1 KO mice congenic on the C57BL/6J background, the strain of choice in many behavioral tests, also showed an improved cognitive phenotype resisting the spatial memory impairments observed in many aged control mice, this time observed in the standard reference memory watermaze and also Y-maze spatial recognition tasks (Yau et al., 2007). Even a 50% reduction in 11β-HSD1 in heterozygous 11β-HSD1 KOs is enough to prevent age-related spatial memory impairments (Sooy et al., 2010).

### Increased hippocampal 11β-HSD1 and impaired spatial memory

Aged C57BL/6J mice showed increased 11β-HSD1 expression in the cortex (layer V) and CA3 cells of the hippocampus compared to young controls. Moreover, hippocampal and cortical 11β-HSD1 mRNA levels correlate with impaired spatial learning in the watermaze (Holmes et al., 2010). The selective increase of 11β-HSD1 in the CA3 subregion of the memory-impaired hippocampus may be of functional significance since it is the CA3 cells that undergo dendritic atrophy following chronic restraint stress or corticosterone injections (Magarinos and McEwen, 1995), both of which impair spatial memory (Luine et al., 1994; Conrad et al., 1996; Wright and Conrad, 2005; Hoffman et al., 2011). CA3 appears crucial for memory acquisition and consolidation in the watermaze (Florian and Roullet, 2004). Furthermore, CA3 cells selectively fail to rapidly encode new spatial information in memory-impaired aged rats (Wilson et al., 2005). This suggests that the implied increase of GC levels driven by variable overexpression of 11β-HSD1 in CA3 of aged mice may be a major contributor to the spatial memory deficits with aging. The mechanisms underlying the upregulation of 11β-HSD1 in CA3 and cortex of aged mice are unknown. GCs and stress elevate hippocampal 11β-HSD1 in young animals affording a possible feed-forward system to amplify GC action (Low et al., 1994). Consistent with increased 11β-HSD1 levels in the hippocampus impairing memory with aging, transgenic mice overexpressing 11β-HSD1 in the forebrain under the CAMIIK promoter resulting in 50% increase in the hippocampus, developed premature memory impairments with deficits in hippocampus-dependent learning tasks (watermaze spatial reference memory and passive avoidance memory) at 18 months (Holmes et al., 2010).

## Hippocampal plasticity and aging

### LTP and LTD

In the absence of hippocampal neuron loss, age-related spatial learning deficits in rats may be the result of more subtle changes in synaptic structure or function (Rapp and Gallagher, 1996; Smith et al., 2000). Consistent with this idea, impaired hippocampal synaptic plasticity, specifically LTP in aged rats relates to individual differences in spatial learning ability (Deupree et al., 1991; Bach et al., 1999). Indeed, LTP induced in the CA1 region using theta-frequency stimulation (5 Hz) was selectively impaired in hippocampal slices from a subpopulation of aged rats that had previously shown poor spatial learning in the water maze (Tombaugh et al., 2002). Elevated corticosterone levels (presumably occupying both MR and GR) also impairs Primed Burst Potentiation (PBP), a low threshold form of LTP, in the hippocampal CA1 in the rat, while low levels of corticosterone (that would occupy mostly MRs) facilitates PBP (Diamond et al., 1992). Although the involvement of other hormones and/or brain regions may also play a role, high levels of corticosterone directly impairs hippocampal synaptic potentiation (Alfarez et al., 2002). In addition, induction of long-term depression (LTD), which in contrast to LTP weakens rather than strengthens synaptic contacts by repeated stimulation (Bear and Malenka, 1994), is enhanced by high corticosterone levels (Coussens et al., 1997) and during aging (Norris et al., 1996, 1998).

### Primed burst potentiation

Primed burst stimulation induces lower potentiation in the hippocampus of young animals compared to the LTP induction protocol (Diamond et al., 1994, 1996; Alfarez et al., 2002), but few reports have shown this reliably in aged animals (Moore et al., 1993). We found very little PBP (a single 200 Hz stimulus followed 180 ms later by a burst of four stimuli at 200 Hz) in hippocampal slices from either 25 months old 11β-HSD1 KO or age matched controls. However, LTP induced with a single 100 Hz tetanus, was increased in hippocampal slices from aged 11β-HSD1 KO mice compared to aged-matched C57BL/6J controls (Yau et al., 2007). Thus, an increase in LTP in the hippocampus of aged 11β-HSD1 KO mice may, in part, underlie their retention of spatial memory with age.

## Glucocorticoids, neurogenesis, and aging

Adult neurogenesis, the generation of new neurons via mitotic cell division, occurs in the dendate gyrus of the hippocampus throughout life. Although the full functional significance of these new neurons is not fully understood, there is increasing evidence to support the notion that these newborn neurons can mature, form synapses, integrate with the local circuitry, and are involved in hippocampus-dependent learning (Lemaire et al., 2012; Marin-Burgin and Schinder, 2012). Of relevance to aging, high levels of GCs or stress reduce neurogenesis (Czeh et al., 2002; Wong and Herbert, 2006). Indeed, neurogenesis is substantially reduced with aging in rodents (Seki and Arai, 1995; Kuhn et al., 1996) and this may be in part related to increased GC levels since within a cohort of aged rats, those with the highest GC levels had the lowest levels of neurogenesis (Montaron et al., 2006). Furthermore, when aged rats were subdivided into aged unimpaired and aged impaired according to their spatial learning abilities (the top and bottom 30% of the population), cell proliferation in the granule cell layer of the dentate gyrus correlated with spatial memory performances (Drapeau et al., 2003). However, when neurogenesis was examined in the aged 11β-HSD1 KO, there was no significant difference compared to aged C57BL/6J controls, although an increase in neurogenesis was observed in young 11β-HSD1 KO mice (Yau et al., 2007). This suggests that the maintained spatial memory in the aged 11β-HSD1 KO mice is not a consequence of increased neurogenesis and that reduced intrahippocampal GCs is insufficient to overcome the other factors linked with aging (e.g., decreased serotonin) that regulate neurogenesis.

## 11β-HSD1 inhibitors and maintenance of spatial memory with aging

Since aged mice with complete or partial 11β-HSD1 deficiency throughout life are protected from spatial memory impairments (Yau et al., 2007; Sooy et al., 2010), inhibiting 11β-HSD1 activity might benefit cognitive function in the aging brain. The big question is can short-term pharmacological inhibition of 11β-HSD1 have memory-enhancing effects in aged rodents and humans? *In vivo* studies in humans are hampered by the non-selectivity of the originally available liquorice-based inhibitors. Carbenoxolone, an old drug formerly used clinically to treat peptic ulcers, inhibits both 11β-HSD1 and 11β-HSD2. This may not matter in the adult brain as the predominant isozyme is 11β-HSD1. Initial small exploratory studies, albeit randomized, double-blind, and placebo-controlled in healthy elderly men and middle-aged patients with type 2 diabetes (52–75 years) showed that carbenoxolone improved aspects of cognitive function (verbal fluency and verbal memory) after 4–6 weeks treatment (Sandeep et al., 2004). Note that amilioride was also given to prevent renal mineralocorticoid excess and hence hypertension. This gave the first indication that inhibition of 11β-HSD1 (assuming the effects were centrally mediated) may be a promising new approach to prevent/ameliorate cognitive decline in humans.

### Selective 11β-HSD1 inhibition

Selective 11β-HSD1 inhibitors that can cross the blood brain barrier have recently been developed (Webster et al., 2007). Two weeks peripheral treatment with a CNS active selective 11β-HSD1 inhibitor (UE1961) in aged C57BL/6J mice improved spatial memory in the Y-maze compared to vehicle treated age-matched controls (Sooy et al., 2010). Moreover, intracerebroventricular administration of another selective 11β-HSD1 inhibitor (UE2316) for 2 weeks also reversed spatial memory impairments in aged C57BL/6J mice confirming mediation by brain 11β-HSD1 inhibition (Yau et al., unpublished). Thus, spatial memory impairments in aged mice are not always a consequence of irreversible brain structural changes and the effects of the inhibitor are most likely the consequence of reduced intracellular GC levels during spatial learning and recall. Previous studies have shown that it is the consequence of increased GCs as a result of hippocampal structural changes (e.g., chronic stress-induced dendritic atrophy) impairing HPA axis feedback that has the more important influence on spatial memory performance since such impairments can be prevented on the day of testing by blocking GC synthesis (Roozendaal et al., 2001; Wright et al., 2006).

### Other potential effects of 11β-HSD1 inhibitors

Depending on the duration of treatment, selective 11β-HSD1 inhibitors may result in resistance of target tissues to GCs, including HPA axis regulatory centers of the brain and pituitary. This could lead to compensatory activation of the HPA axis in an attempt to compensate for the GC deficiency in negative feedback loci expressing 11β-HSD1 in a similar manner to that observed in 11β-HSD1 KO mice on the 129 genetic background (Harris et al., 2001). A hyperactive HPA axis would lead not only to excess plasma cortisol, but perhaps also to excess mineralocorticoid precursors (corticosterone and deoxycorticosterone) and androgens, all of which may manifest as unwanted side effects, such as hypertension and hirsutism/hyperandrogenization in women. The degree of HPA axis activation may depend on the genetic background of the individual, as in mice, as well as the levels of 11β-HSD1 in these tissues, which may differ under various environmental conditions (e.g., with stress, diet, aging). There is also the question of whether inhibition of 11β-HSD1 will affect other types of memory other than spatial memory in cognitively impaired aged individuals. In particular, emotional and fear associated memories, involving the basolateral amygdala, are known to be enhanced with increased GCs (Roozendaal and McGaugh, 2011). Hence, 11β-HSD1 inhibitors may compromise the strength of such memories but whether this is the case remains to be determined.

## Maintenance of spatial memory with aging via predominant MR activation

Impaired spatial memory in aged C57BL/6J mice correlates with higher plasma GCs which are thought to activate the lower affinity GR. Indeed central blockade of GR (but not MR) for 2 weeks reversed the impaired spatial memory in aged C57BL/6J mice in the Y-maze (Yau et al., 2011). This again suggests that the impaired memory in aged C57BL/6J mice is not a direct consequence of irreversible structural changes. The improved spatial memory in aged 11β-HSD1 KO mice has been proposed to occur via reduced intracellular GC levels altering the balance of receptor activation in favor of MR activation. In support, central blockade of MR but not GR in aged 11β-HSD1 KO mice reversed their improved spatial memory phenotype such that they were now impaired in the Y-maze (Yau et al., 2011). Therefore, decreasing GR/increasing MR activation appears effective at preventing spatial memory impairments even in already aged mice. Reducing GC action by altering the receptor balance from predominant GR activation to predominant MR activation may be achieved by the use of selective 11β-HSD1 inhibitors or GR antagonists. Long-term treatment with GR antagonists in humans, however, may potentially cause compensatory activation of the HPA axis to overcome the blockade thus producing generalized GC resistance (Bamberger and Chrousos, 1995). While the use of selective 11β-HSD1 inhibitors causes compensatory activation of the HPA axis simply to accommodate the reduced regeneration of GCs, elevation of cortisol levels was not observed with carbenoxolone in elderly humans (Sandeep et al., 2004) or with selective 11β-HSD1 inhibitors in clinical trials (Rosenstock et al., 2010). Similarly, corticosterone levels were not increased in aged C57BL/6J mice treated short-term with a selective 11β-HSD1 inhibitor (Sooy et al., 2010).

### Links between cognition and glucose metabolism

GCs play a role in the regulation of peripheral glucose mobilization and metabolism. Much less is known about the effects of GCs on glucose metabolism in brain tissues. GCs inhibit glucose utilization in the hippocampus (Sapolsky, 1986). Adrenalectomy decreases serum glucose levels and increases hippocampal glucose utilization (Kadekaro et al., 1988). While exogenously administered GCs adversely affect human memory consolidation and recall (Newcomer et al., 1994), glucose improves memory performance in normal elderly individuals (Hall et al., 1989) and in subjects with probable Alzheimer's disease (Craft et al., 1993). Studies in rodents suggest that hyperglycemia and high-fat diets adversely affect hippocampal function (Kamal et al., 2000; Molteni et al., 2002). In humans, hyperglycemia associate with decline in cognitive function with aging (Convit et al., 2003). Since 11β-HSD1 KO mice lack the enzyme in all tissues, it is possible that the cognitive protection in aged 11β-HSD1 KO mice may reflect, in part, their improved metabolic profiles (Kotelevtsev et al., 1997; Morton et al., 2001), rather than solely the direct effects of enzyme deficiency in the brain. However, aged 11β-HSD1 KO mice show no significant difference in glucose tolerance compared to age-matched controls (Yau et al., 2007). Moreover, the 11β-HSD inhibitor carbenoxolone improved cognitive function in aged humans without altering plasma glucose levels (Sandeep et al., 2004). Thus, the implication is that direct CNS effects of 11β-HSD1 deficiency or inhibition are more likely responsible for the observed improved cognitive function.

## Hippocampal gene expression associated with 11β-HSD1 deficiency and maintenance of spatial memory with aging

To further our understanding of how GC action affects age-related memory performance, it will be important to determine the downstream genes/pathways activated by hippocampal MR and GRs that leads to the impaired and improved spatial memory of aged mice with and without 11β-HSD1. Several studies have examined hippocampal gene expression changes associated with age-related cognitive decline in rats and mice (Burger et al., 2007; Rowe et al., 2007; Pawlowski et al., 2009). Using microarrays, many hippocampal genes were found to be altered with aging regardless of cognitive status. Down-regulated genes in rat hippocampus are involved in mitochrondrial function, cell signaling, neural plasticity, and synaptic function whereas up-regulated genes underpin inflammation, glial structure, cholesterol transport, and lipid/protein degradation. Genes selectively downregulated in spatial memory impaired aged rats characterized in the watermaze and culled soon after training include a number of immediate early genes (IEGs) such as *Arc and Zif268* (also called *Egr-1* and *NGFI-A*) and glucose ultilization/insulin signaling genes such as *Irs1, GcK*, and *Insr* (Rowe et al., 2007). *Zif268*, is thought to activate the transcription of genes essential for hippocampus-dependent long-term memory (Jones et al., 2001). Reduced resting levels of *Zif268* mRNA in the CA1 hippocampal area of aged rats with impaired spatial learning have been observed by microarray analysis and in situ hybridization histochemistry (Yau et al., 1996; Blalock et al., 2003). Whether the selective alterations in gene transcription in cognitively impaired but not unimpaired aged animals is related to their HPA activity and hence circulating GC levels is not known. Hippocampal gene expression microarrays and proteomic approaches in behaviorally defined aged mice including aged 11β-HSD1 KO mice and animals given selective 11β-HSD1 inhibitors may indicate new drug targets for the enhancement of memory in the aged individual.

## Neurosteroids, CYP7B1, and spatial memory with aging

**Figure 5:**
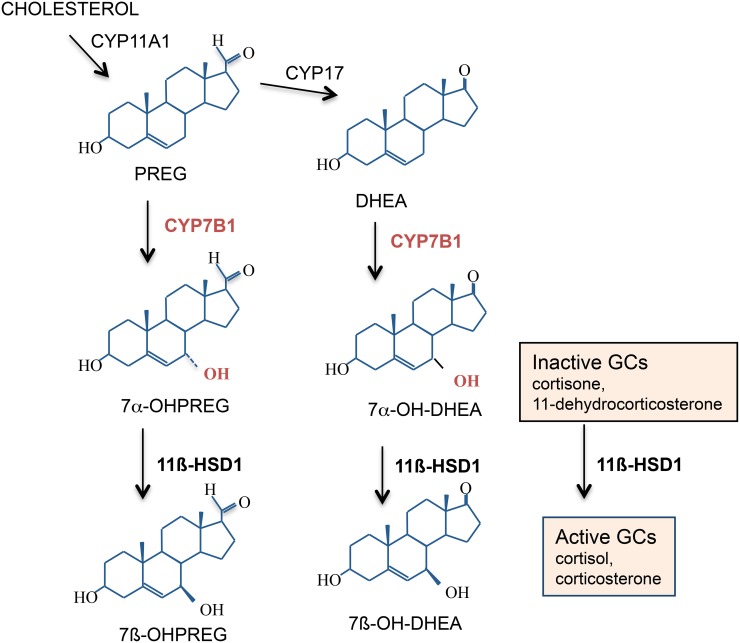
**7α-Hydroxylated steroids are also substrates for 11β-HSD1.** Both 11β-HSD1 and CYP7B1 are expressed in the rodent and human hippocampus. CYP7B1 7α-hydroxylates pregnenolone (PREG) and dehydroepiandrosterone (DHEA) into their more active 7α-hydroxylated metabolites which are also substrates for 11β-HSD1. If levels of these 7α-hydroxylated metabolites within specific cells become high enough, they may compete with the inactive GCs for 11β-HSD1 and effectively act as endogenous “inhibitors” of 11β-HSD1 amplification of intracellular GC action.

Recent studies have revealed additional substrates for 11β-HSD1. These include additional functions in the metabolism of neurosteroids such as dehydroepiandrosterone (DHEA) and pregnenolone, metabolism of 7-oxysterols, as well as in the detoxification of various xenobiotics (Odermatt and Nashev, 2010). Neurosteroids have long been implicated to play an important role in the modulation of spatial learning and memory processes (Vallee et al., 2001). Circulating levels of DHEA derive from the adrenal cortex and decline significantly with aging in humans and this parallels cognitive decline (Orentreich et al., 1992). In rodents there is very little DHEA in brain because they lack adrenal expression of the enzyme cyp17α that converts pregnenolone to DHEA (Le Goascogne et al., 1991) though there may be limited local CNS synthesis (Liu et al., 2009). However, high levels of its precursor pregnenolone have been measured in the hippocampus of young rats and this declines with aging correlating with spatial memory impairments (Vallee et al., 1997). Pregnenolone, DHEA, and related 3β-hydroxysteroids are 7α-hydroxylated in brain by the cytochrome P450-7B1 (CYP7B1) enzyme into their bioactive steroid metabolites (Rose et al., 1997). High levels of CYP7B1 is expressed in the hippocampus and enzyme activity is selectively decreased in aged rats with impaired spatial memory but not in aged cognitively unimpaired rats (Yau et al., 2006). In the memory-impaired aged rats, central administration of an active product, 7α-hydroxypregnenolone, reversed the spatial memory deficits and improved memory in a radial arm watermaze memory task thus effectively overcoming reduced CYP7B1 activity in the aged brain (Yau et al., 2006). It has recently been proposed that the products of CYP7B1, 7α-hydroxypregnenolone, and 7α-hydroxyDHEA, may exert anti-GC effects in target tissues by competing with 11-keto GCs for access to 11β-HSD1, thus attenuating regeneration of active GCs (Muller et al., 2006a). Thus, 11β-HSD1, in addition to its known role in reactivating GCs within target cells, has also been shown to carry out the inter-conversion of 7α-hydroxyDHEA into 7β-hydroxyDHEA, at least *in vitro* (Muller et al., 2006b) (Figure 5). Enzyme kinetic data from yeast-expressed human 11β-HSD1 implies that the 7-hydroxysteroid substrates are preferred to cortisone. Hence in tissues, 7-hydroxysteroid substrates may act like endogenous “inhibitors” of 11β-HSD1, reducing their regeneration of active GCs. This adds another level of potential fine tuning of GC action within specific target CNS cells but whether this occurs *in vivo* under normal or pathological conditions is not known.

## Conclusions

The local amplification of GC action in the brain by 11β-HSD1 plays a pivotal role in the emergence of spatial learning impairments with aging. Evidence over the past decade has confirmed that while elevated plasma GCs correlate with impaired spatial learning with aging, it is the level of active GCs within specific brain cells regulated by 11β-HSD1 that appears essential for the control of spatial memory in the aged animal. Importantly, whereas treatments to lower blood GC levels need to be maintained long-term to ameliorate spatial memory deficits in aged rats, lowering GC levels inside cells expressing the enzyme by selective 11β-HSD1 inhibitors requires only short-term treatment and can reverse spatial memory deficits in already aged mice. Whether this is the case in humans remains to be tested. The data also show that spatial memory deficits in aged rodents are not necessarily irreversible due to structural changes but appear to be regulated in the short-term by intracellular GC actions activating MR and GR to regulate the transcription of target genes that influence memory formation. Future investigations to determine the mechanisms whereby 11β-HSD1 in the brain is upregulated by aging and the downstream pathways following predominant brain MR activation in aged 11β-HSD1 KO mice will be crucial in the understanding of how GCs maintain or impair hippocampus-dependent spatial learning and memory with aging. Whether or not this approach is of utility in pathological brain aging, such as Alzheimer's disease and other dementias, remains to be determined, but of substantial interest.

### Conflict of interest statement

The authors declare that the research was conducted in the absence of any commercial or financial relationships that could be construed as a potential conflict of interest.
